# Cohort profile: the Viral load Cohort North-East Lesotho (VICONEL) from 2016 to 2023 – cohort description, test volumes, predictors of viraemia and the road ahead

**DOI:** 10.1136/bmjopen-2024-085404

**Published:** 2025-02-06

**Authors:** Jennifer Anne Brown, Lipontso Motaboli, Malebanye Lerotholi, Maurus Kohler, Kathrin Haenggi, Moliehi Mokete, Makobefo Gladys Chakela, Mpho Kao, Mathebe Kopo, Moleboheng Mokebe, Lorena Urda, Bienvenu Lengo Nsakala, Blaise Lukau, Irene Ayakaka, Alain Amstutz, Jochen Ehmer, Anna Klicpera, Thomas Klimkait, Tracy Glass, Josephine Muhairwe, Frédérique Chammartin, Nadine Tschumi, Niklaus Daniel Labhardt

**Affiliations:** 1 Division of Clinical Epidemiology, Department of Clinical Research, University Hospital Basel, Basel, Switzerland; 2 University of Basel, Basel, Switzerland; 3 SolidarMed, Partnerships for Health, Maseru, Lesotho; 4 Ministry of Health of Lesotho, Maseru, Lesotho; 5 Oslo Centre for Biostatistics and Epidemiology, Oslo University Hospital and University of Oslo, Oslo, Norway; 6 Bristol Medical School, Population Health Sciences, University of Bristol, Bristol, UK; 7 SolidarMed, Partnerships for Health, Lucerne, Switzerland; 8 Molecular Virology, Department of Biomedicine, University of Basel, Basel, Switzerland; 9 Department of Medicine, Swiss Tropical and Public Health Institute, Basel, Switzerland

**Keywords:** HIV & AIDS, INFECTIOUS DISEASES, PUBLIC HEALTH, Epidemiology

## Abstract

**Abstract:**

**Purpose:**

The prospective Viral load Cohort North-East Lesotho (VICONEL) aims to support clinical management and generate scientific evidence to inform HIV care. Specifically, VICONEL allows for the monitoring of HIV treatment outcomes and health system performance, encompasses a biobank for further research with routinely collected blood plasma samples of consenting participants and provides a valuable framework for nested observational and interventional studies.

**Participants:**

VICONEL captures routine viral load test results alongside associated demographic and treatment information among people in care for HIV in Lesotho, southern Africa. As of December 2023, it encompasses all viral load testing from 24 healthcare facilities in two districts of Lesotho.

**Findings to date:**

From January 2016 to December 2023, 137 660 viral load test results were available for 29 380 participants. At the time of the last viral load test, median age was 42 years (IQR: 33–53); 18 511 (63%) were adult women, 10 029 (34%) adult men and 835 (3%) children <15 years (age/sex missing for 5) and median time taking antiretroviral therapy (ART) was 6.4 years (IQR 3.2–9.9). Overall, the proportion of cohort participants with viral suppression to <1000 copies/mL has continually exceeded 90% and has been above 95% since 2020; however, this proportion has consistently been lower among children. Sex, age category/ART regimen core agent (combined variable), time since ART initiation and district were independently associated with viraemia.

**Future plans:**

VICONEL offers potential for (1) further digitalisation and automation of results sharing at the client, facility and district/national level, (2) integration of additional clinical and diagnostic data, including comorbidities and drug resistance and (3) embedding randomised trials.

**STRENGTHS AND LIMITATIONS OF THIS STUDY**

- VICONEL (Viral load Cohort North-East Lesotho) covers all HIV viral load testing from 24 clinics in two districts in Lesotho and is thus highly representative.
- Core functions can be maintained at low cost, constituting a model for near-real-time monitoring of treatment outcomes with limited resources.
- Data capture occurs at the time point of viral load testing; thus, treatment or clinical data are not updated between viral load tests, and reasons for exiting the cohort are not followed up. Participant data beyond viral load results are limited to key demographic, clinical and treatment information.
- The cohort and associated biobank have proven to be a valuable platform for nested observational and interventional research, including randomised trials.
- The cohort will be of high value to monitor emerging HIV resistance to newer antiretroviral drugs, such as dolutegravir.

## Introduction

 Prospective cohorts provide longitudinal data to monitor services and health outcomes within HIV programmes. This facilitates the identification of gaps in service delivery and uptake, early detection of emerging public health threats and assessments of temporal trends or effects of guideline changes, potentially informing future guidelines.^1 2^ Furthermore, they can serve as a platform for nested randomised clinical trials using the routinely collected data for recruitment and endpoint collection.

Regular viral load testing is essential to monitor the effectiveness of antiretroviral therapy (ART) for people with HIV and to take timely action if required. Indeed, the WHO has recommended viral load testing as the preferred method of monitoring treatment outcomes since 2013.^3^ In Lesotho, southern Africa, viral load monitoring began shortly after these recommendations but was initially centralised in the capital city, Maseru. Starting in 2015, a consortium consisting of the Ministry of Health of Lesotho, the District Health Management Team of Butha-Buthe, the non-profit organisation SolidarMed, the Swiss Tropical and Public Health Institute and the University of Basel collaboratively supported the District Laboratory of Butha-Buthe Government Hospital in northern Lesotho to launch the first decentralised viral load testing platform in the country. At the time, access to viral load monitoring in the periphery was severely restricted due to limited capacity for centralised testing and the need for long-distance transport of samples and paper-based results. The decentralised viral load testing laboratory and an associated viral load database were launched in December 2015 and officially inaugurated by the Minister of Health in June 2016. After 6 months of piloting service provision for Butha-Buthe Government Hospital, viral load testing through this laboratory was gradually rolled out to all clinics in Butha-Buthe district and, from 2018 onwards, to neighbouring Mokhotlong district. In conjunction with building the laboratory infrastructure and database, the Viral load Cohort North-East Lesotho (VICONEL) was established to monitor viral load outcomes among persons with HIV who are in care in Mokhotlong or Butha-Buthe district.

Here, we report on cohort composition, test numbers, treatment outcomes and factors associated with viraemia from the first 8 years of the cohort (2016–2023), review the cohort’s broader scientific findings so far and outline the road ahead.

## Cohort description

### Cohort design

VICONEL was designed in conjunction with the establishment of decentralised viral load testing in Butha-Buthe, Lesotho. The prospective open cohort includes people with HIV who receive viral load monitoring with samples tested at the laboratory of Butha-Buthe Government Hospital or, since 2020, through near-point-of-care viral load monitoring currently available at three participating clinics (Seboche Mission Hospital, Mokhotlong Government Hospital, Mapholaneng Health Centre). It includes people in care in Butha-Buthe district since the start of the cohort and people in care in Mokhotlong district since 2018. As of December 2023, 3 hospitals, 19 peripheral nurse-led health centres, 1 private clinic and 1 clinic for mine workers were included in the cohort. The cohort study includes a biobank storing leftover plasma samples from viral load testing.

### Objectives

VICONEL aims to support local clinical management of HIV and generate scientific evidence to inform local and global HIV care. The former is achieved in part through a dashboard for clinicians and automated reports flagging persons with incident or repeated viraemia to facilitate clinical follow-up. Scientific evidence is generated through analysis of routine data,^4^,^8^ as well as nested observational^9^,^12^ and interventional studies.^13^

### Setting

At 18.5%, Lesotho has the second highest adult HIV prevalence among all countries globally.^14^ In 2023, 270 000 people in the country were living with HIV and 4 800 people newly acquired HIV.^14^ The majority of the population lives rurally, impeding access to healthcare. This is further compounded by the scarcity of medical professionals, with just 4.7 medical doctors and 32.6 nursing and midwifery professionals per 10 000 population.^15^

VICONEL covers 2 of the 10 districts in the country with a combined population of around 220 000 people.^16^ Previous analyses within the cohort have demonstrated serious gaps along the viral load care cascade for children and adults with HIV in this setting.^4 5^

Beyond the progressive rollout of decentralised viral load testing (and the VICONEL cohort), the rollout of ART containing the integrase strand transfer inhibitor (INSTI) dolutegravir as a core agent has constituted another key change in care provision during the reporting period. Dolutegravir rollout began in Lesotho in late 2019, with the main programmatic transition from non-nucleoside reverse transcriptase inhibitor (NNRTI)-based to dolutegravir-based ART occurring throughout 2020. For children taking ART containing the protease inhibitor (PI) ritonavir-boosted lopinavir as the core agent, the main transition period to dolutegravir-based ART took place in 2022 and 2023.

### Procedures

For the period 2016–2023, according to national guidelines, adults with HIV should receive viral load testing six and 12 months after starting ART or switching to second-line or third-line ART, as well as annually thereafter, whereas 6 monthly testing is recommended for children and adolescents (0–19 years).^17 18^ More frequent viral load testing is indicated during pregnancy and breastfeeding, as well as on detection of viraemia. Specifically, viraemia ≥1000 copies/mL or, since 2022, ≥50 copies/mL should trigger several sessions of enhanced adherence counselling followed by repeat viral load testing. For NNRTI-based first-line ART, a switch to second-line ART was indicated in case of sustained viraemia. For sustained viraemia with PI-based or INSTI-based ART, national guidelines recommend consulting the ART advisory committee and considering genotypic resistance testing.^17 18^ However, genotypic resistance testing and subsequent treatment switches require centralised approval, are relatively uncommon, and resistance results are currently not routinely transferred to the VICONEL database.

Blood samples collected in healthcare facilities in Butha-Buthe or Mokhotlong districts are transported to one of the three hospital laboratories, typically by a motorbike transport service, for plasma separation. A national Viral Load Request Form is filled out by the treating facility and transported with the sample. On reception at the hospital laboratory, data are entered into the Laboratory Information System (LIS) managed by the Ministry of Health. If the sample is first received in one of the two other hospital laboratories, the separated plasma is then transported to the Butha-Buthe Government Hospital laboratory. Viral load testing was initially performed using a COBAS Ampliprep/COBAS TaqMan HIV-1 Test, V.2.0, and later using the COBAS 4800 system HIV-1 Test (F. Hoffmann-La Roche AG, Basel, Switzerland). Samples are linked to the instrument position via barcode scanning before viral load testing. Once the run is complete and the testing laboratory has authorised results, viral load results feed into the LIS, on which the referring laboratories can review data and paper-based results forms can be printed and distributed to the respective facilities. Data are extracted from the local LIS approximately weekly and uploaded to the VICONEL database maintained by SolidarMed and the Division of Clinical Epidemiology, University Hospital Basel. The data team of SolidarMed Lesotho then confirms each viral load result and associated information, cross-checking the Viral Load Request Form against existing data in the database and updating the database information (eg, ART regimen, regimen start and stop dates) if applicable. If necessary, healthcare facilities are contacted to complete missing or unclear information. In addition, the VICONEL data manager runs data quality checks on a regular basis and investigates unusual entries. Data routinely collected in the cohort database include demographic, clinical, laboratory, treatment, consenting and biobank data.

In addition to laboratory-based testing, since 2020, a small proportion of people with HIV have received near-point-of-care viral load testing using Xpert HIV-1 Viral Load on GeneXpert Systems (Cepheid, Sunnyvale, California, USA), available at two hospitals and one health centre. According to current guidelines, point-of-care viral load testing is prioritised for infants, children, adolescents, pregnant and breastfeeding people and people with HIV viraemia.^18^ Currently, viral load results from GeneXpert Systems are not integrated into the LIS, necessitating manual collection of associated participant data from Viral Load Request Forms and local registries.

Plasma samples left over after viral load testing are stored in a biobank for clinical and, if informed consent for further use is provided, research purposes. At the time of writing, this is operationally limited to viraemic samples.

The cohort does not interfere with the scheduling of viral load testing. However, its database is used, notably by local clinicians, to support clinical management.

### Analyses and statistical methods

The data presented here include viral load results from 1 January 2016 to 31 December 2023. We exclude entries (1) where the viral load result is missing (ie, failed tests); (2) where viral load testing occurred before or on the day of the first-ever ART initiation (not part of routine care in Lesotho); (3) where both the date of blood draw and the date of testing are missing; or (4) from unknown facilities, facilities contributing less than 50 viral load results throughout the reporting period, referring facilities outside of Butha-Buthe or Mokhotlong district, or from Mokhotlong district before 1 January 2018. If multiple viral load tests are conducted for the same individual on the same day (eg, due to suspected testing errors), the highest viral load is considered. We include individuals with at least one available viral load result after the application of these criteria (online supplemental figure S1).

We report on cohort composition, viral load testing and turnaround time (defined here as time from blood draw to viral load testing) and the proportion of participants with viraemia by year for the entire cohort and by district. Ages <15 years are defined as children and ages ≥15 years as adults, in line with UNAIDS reporting.^14^ In addition, we assessed for factors associated with viraemia ≥50 copies/mL or ≥1000 copies/mL at the level of the individual viral load result using a mixed-effect logistic regression model with the participant as the random effect, excluding observations with missing data for any of the independent variables. For this analysis, age category and ART core agent were represented in a single variable due to collinearity. When the core agent was changed immediately after a documented viral load ≥50 copies/mL or ≥1000 copies/mL, respectively, viral load results following this regimen change were excluded to avoid misleading interpretation, as switching treatment due to viraemia represents a unique opportunity for resuppression within an individual.

All statistical analyses were performed using Stata MP V.16.1.

## Findings to date

### Participant characteristics over time

Overall, 29 380 individuals with HIV received at least one valid viral load result through this platform from January 2016 through December 2023 and were thus included (table 1; online supplemental table S1). Of those with at least one viral load result in 2023, 12 285/19 294 (64%, sex missing for one participant) were female, the median age was 44 years (IQR 35–55), 597/19 295 (3%) were children <15 years of age, median time since ART initiation was 7.1 years (IQR 4.0–11.0) and the most common ART regimen was tenofovir disoproxil fumarate/lamivudine/dolutegravir, prescribed to 17 526/19 295 (91%) participants. The number of individuals receiving at least one viral load test in any given year increased in line with initial cohort expansion, with a particular jump in 2018 as Mokhotlong district was included. Median follow-up time from the first to the last included viral load result was 3.5 years (IQR 1.0–5.5); during this time, a median of 4 (IQR 2–7) viral load results were recorded (table 1; online supplemental table S1).

**Table 1:** Participant characteristics (2017 – 2023)

	2017	2018	2019	2020	2021	2022	2023	Total (latest viral load within 2016–2023)
N	8666	13 492	15 216	17 439	17 695	18 530	19 295	29 380
Year of blood draw for last viral load, median (IQR)	–	–	–	–	–	–	–	2023 (2022–2023)
Years from first to last included viral load, median (IQR)	–	–	–	–	–	–	–	3.5 (1.0–5.5)
Number of viral loads per individual, median (IQR)	–	–	–	–	–	–	–	4 (2–7)
District, n (%)								
Butha-Buthe	8666 (100%)	8840 (66%)	10 164 (67%)	10 626 (61%)	10 985 (62%)	11 103 (60%)	11 738 (61%)	18 165 (62%)
Mokhotlong	0 (0%)	4652 (34%)	5052 (33%)	6813 (39%)	6710 (38%)	7427 (40%)	7557 (39%)	11 215 (38%)
Health facility type, n (%)								
Hospital	5168 (60%)	7074 (52%)	7623 (50%)	8053 (46%)	8039 (45%)	8373 (45%)	8660 (45%)	13 452 (46%)
Health centre	3218 (37%)	6100 (45%)	7211 (47%)	9018 (52%)	9216 (52%)	9791 (53%)	10 308 (53%)	15 269 (52%)
Other	280 (3%)	318 (2%)	382 (3%)	368 (2%)	440 (2%)	366 (2%)	327 (2%)	659 (2%)
Age, median (IQR)*	41 (32–52)	42 (33–52)	42 (33–52)	42 (33–53)	43 (34–53)	44 (35–54)	44 (35–55)	42 (33–53)
Age and sex, n (%)†								
Female adult (≥15 years)	5590 (65%)	8534 (63%)	9737 (64%)	10 717 (61%)	10 916 (62%)	11 557 (62%)	11 975 (62%)	18 511 (63%)
Male adult (≥15 years)	2629 (30%)	4237 (31%)	4749 (31%)	5953 (34%)	6087 (34%)	6346 (34%)	6722 (35%)	10 029 (34%)
Female child (<15 years)	239 (3%)	360 (3%)	372 (2%)	383 (2%)	351 (2%)	318 (2%)	310 (2%)	439 (1%)
Male child (<15 years)	206 (2%)	359 (3%)	356 (2%)	385 (2%)	341 (2%)	309 (2%)	287 (1%)	396 (1%)
Year of first recorded ART start, median (IQR)								2016 (2012–2018)
Years since first recorded ART start, median (IQR)	3.6 (1.5–6.5)	4.0 (2.0–7.4)	4.6 (2.5–8.0)	5.1 (3.0–8.8)	6.0 (3.4–9.6)	6.6 (3.9–10.3)	7.1 (4.0–11.0)	6.4 (3.2–9.9)
ART regimen, n (%)								
TDF+3TC+DTG	0 (0%)	0 (0%)	195 (1%)	7912 (45%)	15 384 (87%)	16 545 (89%)	17 526 (91%)	22 167 (75%)
ABC+3TC+DTG	0 (0%)	0 (0%)	10 (0%)	623 (4%)	954 (5%)	966 (5%)	1138 (6%)	1344 (5%)
AZT+3TC+DTG	0 (0%)	0 (0%)	2 (0%)	141 (1%)	232 (1%)	223 (1%)	241 (1%)	309 (1%)
TDF+3TC+LPV/r	74 (1%)	113 (1%)	129 (1%)	123 (1%)	100 (1%)	98 (1%)	62 (0%)	141 (0%)
ABC+3TC+LPV/r	115 (1%)	207 (2%)	248 (2%)	260 (1%)	252 (1%)	231 (1%)	114 (1%)	202 (1%)
AZT+3TC+LPV/r	144 (2%)	260 (2%)	309 (2%)	255 (1%)	227 (1%)	198 (1%)	140 (1%)	253 (1%)
TDF+3TC+EFV	5788 (67%)	9215 (68%)	10 689 (70%)	7121 (41%)	417 (2%)	193 (1%)	45 (0%)	3903 (13%)
ABC+3TC+EFV	409 (5%)	562 (4%)	605 (4%)	233 (1%)	39 (0%)	18 (0%)	4 (0%)	215 (1%)
AZT+3TC+EFV	1073 (12%)	1684 (12%)	1594 (10%)	421 (2%)	40 (0%)	23 (0%)	4 (0%)	426 (1%)
TDF+3TC+NVP	412 (5%)	477 (4%)	472 (3%)	81 (0%)	11 (0%)	4 (0%)	1 (0%)	132 (0%)
ABC+3TC+NVP	30 (0%)	36 (0%)	29 (0%)	7 (0%)	4 (0%)	1 (0%)	1 (0%)	19 (0%)
AZT+3TC+NVP	593 (7%)	908 (7%)	901 (6%)	239 (1%)	24 (0%)	17 (0%)	2 (0%)	233 (1%)
Other/unknown	28 (0%)	30 (0%)	33 (0%)	23 (0%)	11 (0%)	13 (0%)	17 (0%)	36 (0%)

2016 is omitted for space reasons; the full table is shown in Table S1online supplemental table S1. For each year, cohort participants with at least one viral load test in the respective year are shown. Data refers to the (time point of the) first viral load test of a given individual in the respective year. In the ‘total’ column, data refers to the (time point of the) last available viral load test for each individual.

* Missing for one in 2017, one in 2018, two in 2019, one in 2020 and three in the total.

† Missing for two in 2017, two in 2018, two in 2019, one in 2020, one in 2023 and five in the total.

3TC, lamivudine; ABCabacavirARTantiretroviral therapyAZT, zidovudine; DTG, dolutegravir; EFV, efavirenz; LPV/r, ritonavir-boosted lopinavir; NVP, nevirapine; TDF, tenofovir disoproxil fumarate

### Viral load testing over time

**Figure 1:**
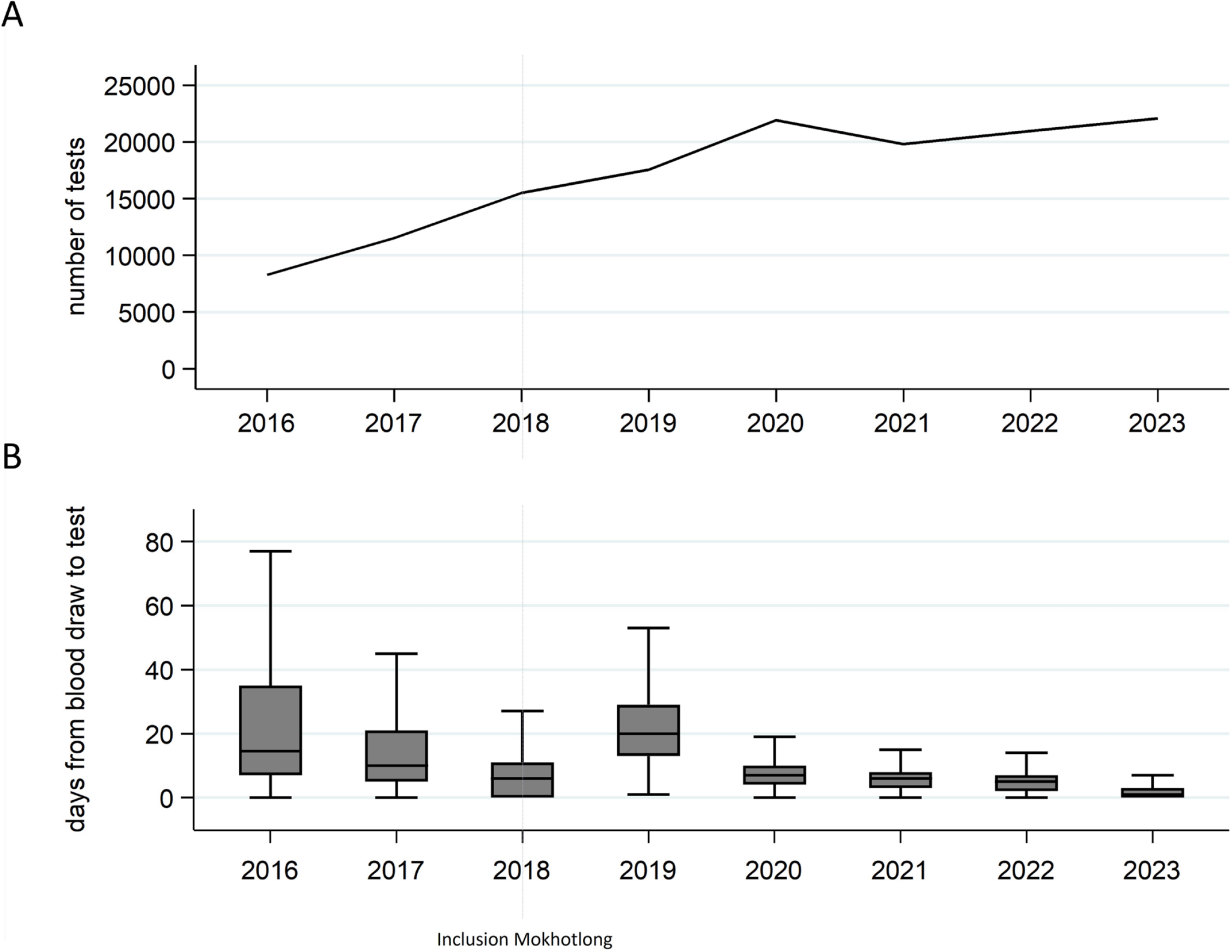
Viral load testing over time. (A) Number of viral load tests by year. (B) Box plots showing turnaround time, defined as the number of days from blood draw to viral load testing, by year (outside values are excluded).

From 1 January 2016 to 31 December 2023, 137 660 viral load tests were conducted. The annual number of samples increased each year until 2020, followed by a slight decrease and stabilisation (figure 1A). The time between blood draw and laboratory-based testing varied greatly by year but overall decreased over time, with a median of 1 (IQR 0–3) days in 2023 (figure 1B). While we did not specifically assess the effect of the COVID-19 pandemic on viral load testing, major disruptions in viral load testing during the pandemic as reported for other settings^19^ are not immediately apparent for this cohort.

### Virological outcomes over time

**Figure 2:**
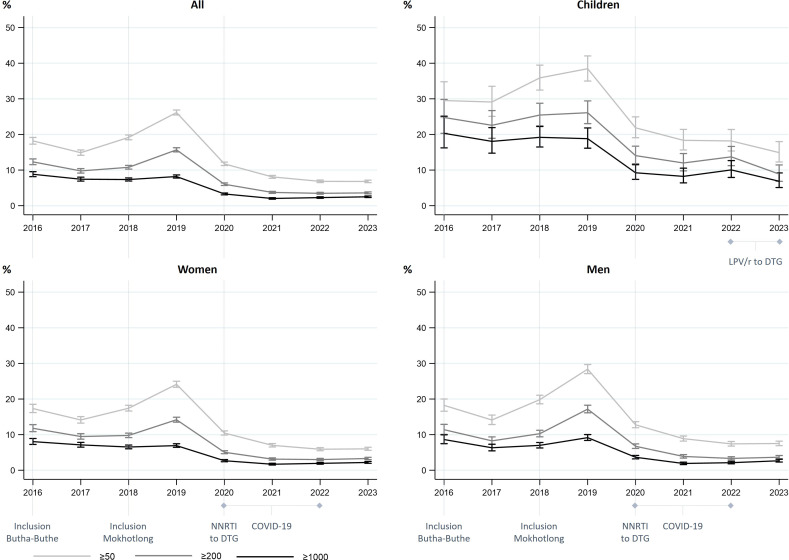
Proportion with viraemia above various thresholds (in copies/mL) over time. The first viral load result of any individual in a given year is considered. The denominator corresponds to the number of individuals receiving at least one viral load test in a given year. Bars indicate 95% CIs. DTG, dolutegravir; LPV/r, ritonavir-boosted lopinavir; NNRTI, non-nucleoside reverse transcriptase inhibitor.

Among people with a viral load in a given year, viral suppression to <1000 copies/mL has consistently exceeded 90% since the launch of the cohort and has exceeded 95% since 2020 (figure 2). Increases in the proportion of participants with viraemia in 2018/2019 might be partially explained by the inclusion of Mokhotlong district from 2018 onwards, as the proportion with viraemia has consistently been higher in Mokhotlong than in Butha-Buthe district (online supplemental figures S2-S3). However, a slight increase in 2019 was also observed in Butha-Buthe, pointing towards additional underlying factors. The subsequently decreased prevalence of viraemia from 2020 onwards appears temporally aligned with the rollout of dolutegravir in Lesotho. Throughout the observation period, viral suppression to <1000 copies/mL among children has been substantially lower than among adults at around 80–82% from 2016 to 2019 and around 90–93% since 2020. This is consistent with reported challenges in achieving viral suppression among children and global reports.^14 20^

Among participants with at least one viral load test in 2023, at their first test in the year, 482/19 295 (2%) had a viral load ≥1000 copies/mL and 1314/19 295 (7%) had a viral load ≥50 copies/mL. Among adult women, adult men and children, 262/11 975 (2%), 179/6722 (3%) and 41/597 (7%), respectively, had viraemia ≥1000 copies/mL and 720/11 975 (6%), 505/6722 (8%), and 89/597 (15%) had viraemia ≥50 copies/mL, respectively (sex missing for one adult).

Among all participants, regardless of the number of recorded viral load results, all viral load results were <50 copies/mL for 18 325/29 380 (62%), at least one was ≥50 copies/mL and all were <1000 copies/mL for 7024/29 380 (24%), and at least one result was ≥1000 copies/mL for 4031/29 380 (14%).

### Factors associated with viraemia

**Figure 3:**
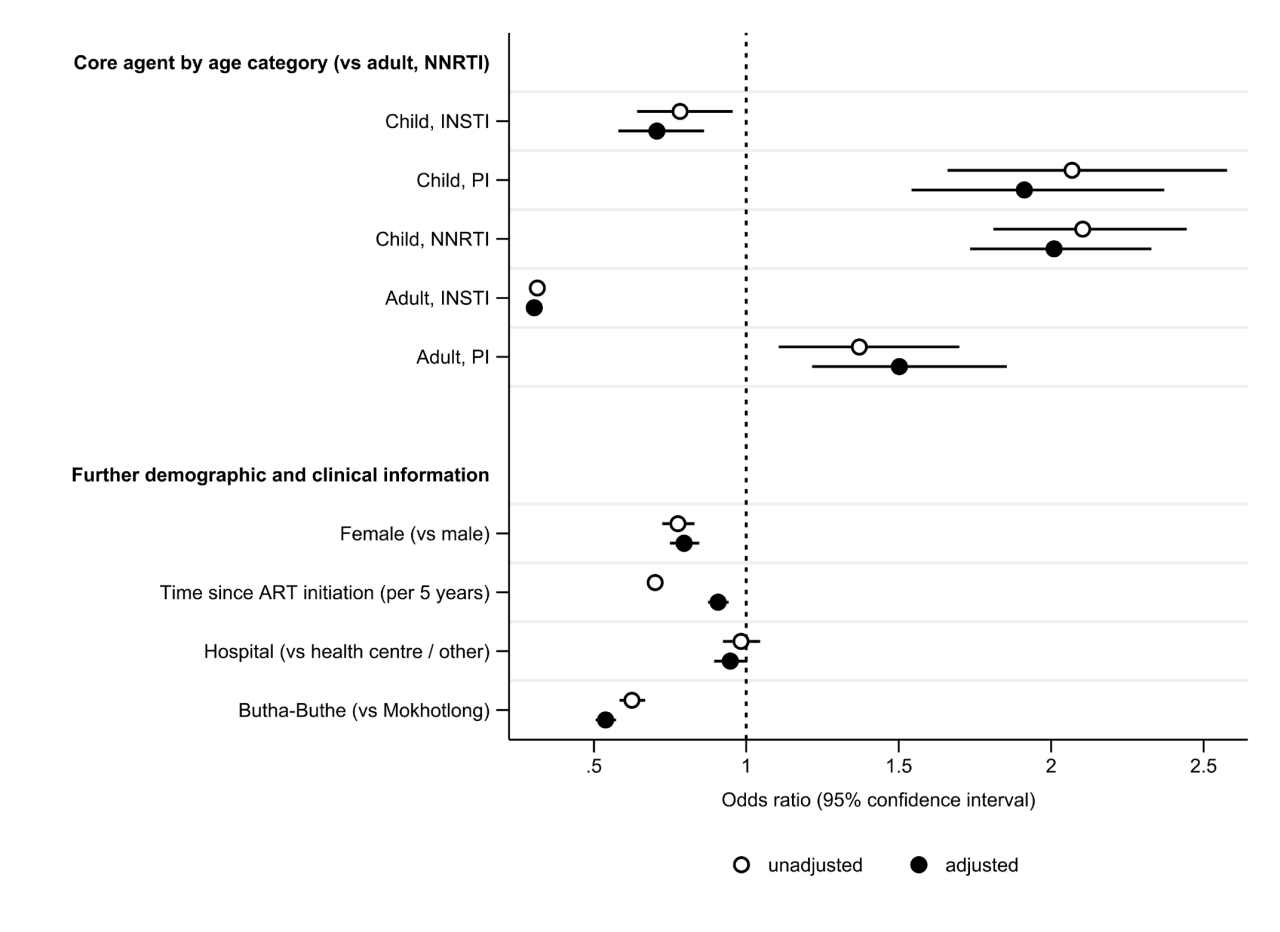
Factors associated with a viral load result ≥50 copies/mL in a logistic regression with mixed effect on the participant level (n=124 663). ORs and 95% CIs of a given viral load result being ≥50 copies/mL are indicated for unadjusted and adjusted analysis. The dotted line at 1 indicates equality of odds. ART, antiretroviral therapy; INSTI, integrase strand transfer inhibitor; NRTI, non-nucleoside reverse transcriptase inhibitor; PI, protease inhibitor.

We assessed factors associated with viraemia using a mixed effects logistical regression model among included viral load results. Being female (vs male; adjusted OR (aOR) 0.80, 95% CI 0.75 to 0.85), increasing time since ART initiation (per 5 years; aOR 0.91, 95% CI 0.87 to 0.94), being in care in Butha-Buthe district (vs Mokhotlong; aOR: 0.54, 95% CI 0.51 to 0.57) and, compared with adults taking NNRTI-based ART, being an adult taking INSTI-based ART (aOR 0.30; 95% CI 0.29 to 0.32) or a child taking INSTI-based ART (aOR 0.71; 95% CI 0.58 to 0.86) were associated with a lower aOR of viraemia ≥50 copies/mL. Being an adult taking PI-based ART (aOR 1.50, 95% CI 1.22 to 1.85), a child taking NNRTI-based ART (aOR 2.01; 95% CI 1.73 to 2.33) or a child taking PI-based ART (aOR 1.91, 95% CI 1.54 to 2.37) were associated with a higher aOR (figure 3). No association with viraemia was observed for receiving care in a hospital (vs health centre or other facility type; aOR 0.95, 95% CI 0.89 to 1.00). The higher aORs for viraemia among children compared with adults taking the corresponding ART regimen aligns with higher overall prevalence of viraemia among children. Higher ORs of viraemia in the context of PIs, especially among adults, may be partly related to their use in second-line ART. Lower odds of viraemia with increasing time since ART initiation was still observed when restricting analysis to either children or adults, though the effect was greater among children; furthermore, restricting analysis to children, no effect of sex was observed (for female (vs male) children: aOR 0.95; 95% CI 0.73 to 1.26).

Increasing the outcome cut-off to ≥1000 copies/mL, some associations of age category and regimen with viraemia were lost though overall results were similar (online supplemental figure S4).

### Review of the cohort’s broader findings to date

Key findings from the VICONEL cohort thus far have highlighted gaps in the management of HIV viraemia, assessed implications of the rollout of dolutegravir, leveraged the cohort biobank to assess serological profiles during the COVID-19 pandemic, and paved the way for an ongoing nested cluster-randomised controlled trial.

First, management of viraemia—that is, timely follow-up viral load testing with switching to second-line ART if indicated—was assessed among children^5^ and adults,^4^ demonstrating major gaps in the viral load care cascade. Among children and adults with a viral load ≥1000 copies/mL, only 28% and 25%, respectively, were considered to be managed in a timely manner according to guidelines. Compared with adult participants in care in hospitals, adults in health centres were more likely to have a viral load ≥1000 copies/mL at the follow-up viral load test (64% vs 44%) and less likely to be switched to second-line therapy in case of sustained viraemia.^4^ In children, delays between a first elevated viral load and confirmatory viral load testing were more pronounced in health centres than in hospitals.^5^

Second, the implications of the large-scale programmatic rollout of dolutegravir-based ART were assessed in children, adolescents and adults. In a nested study within VICONEL, we observed no negative changes in self-reported symptoms among adults changing from efavirenz- to dolutegravir-based ART, with potential moderate improvements observed for questions relating to depressed mood, anxiety and nightmares or strange or vivid dreams.^10^ We found that previously described associations of low-level viraemia (50–999 copies/mL) with subsequent viraemia ≥1000 copies/mL hold true also in the era of dolutegravir, supporting the 2021 change in WHO guidelines lowering the viral load threshold for clinical action.^7 21^ Overall, short-^9^ and long-term viral load outcomes after changing from NNRTI-based to dolutegravir-based ART were encouraging, with over 95% of adults and over 90% of children and adolescents with non-missing data at 24 months having viral suppression to <50 copies/mL.^6 8^ However, our recent findings raise important concerns regarding emergent resistance to dolutegravir: among 85 participants who had persistent and/or recurring viraemia including at least one viral load ≥500 copies/mL at least 18 months after starting dolutegravir, consented to further use of their stored samples, and had available samples in the biobank that were successfully sequenced, dolutegravir resistance was observed for 8 (9%).^22^ All eight participants with dolutegravir resistance were in care at health centres (as opposed to hospitals).

Third, serological responses to endemic human coronaviruses and SARS-CoV-2 during the first 18 months of the COVID-19 pandemic were measured using stored biobank samples. This allowed for more detailed reporting on early pandemic dynamics than had previously been available for Lesotho. We identified associations of female sex and high BMI with SARS-CoV-2 seropositivity and observed positive correlations between the strength of response to endemic human coronaviruses and to SARS-CoV-2.^11^

Finally, formative research on client preferences^12^ helped inform the design of the *VIral load Triggered ART care Lesotho* (VITAL) trial, an ongoing cluster-randomised controlled trial within VICONEL. VITAL aims to reduce waiting times, unburden health centres, and reallocate client and healthcare resources in a needs-driven and preference-driven manner. To do so, the VITAL intervention entails a viral load-driven differentiated service delivery model with automated SMS-based viral load result reporting to participants and an app-based eHealth clinical decision support tool for ART nurses.^13^

### Strengths and limitations

Analyses within VICONEL have several limitations. The database is slim and routinely captures minimal demographic, treatment and clinical data only, whereas data on clinical visits and pharmacy dispensing are not available. This implies that events such as hospitalisations or death are not routinely captured. As data capture occurs only at the time of viral load testing, retention in care cannot be ascertained for those without continued viral load testing.

However, VICONEL also has several strengths. Covering two districts, it is highly representative of people on ART receiving viral load testing in this setting. Data quality is strengthened through obtaining viral load results directly as instrument exports and through multilevel data checks. Aggregate reports and dashboards aim to support clinical management. Operationally, the reliance on routine data collection keeps maintenance costs low while allowing for near-real-time monitoring in a resource-limited setting; furthermore, the existing framework and biobank facilitate the integration of nested non-routine research. Finally, VICONEL has been a successful project for sustainable capacity building: initial laboratory infrastructure and operational costs were first largely funded by research grants. Subsequently, the Ministry of Health has gradually taken over costs for instrument maintenance and replacements, reagents and consumables and partial human resource costs. In December 2023, operational costs (excluding data management and analysis) were limited to salaries for two laboratory technologists and two data clerks. As such, VICONEL constitutes a model for integrated cross-sectional implementation research with a stepwise handover of responsibilities from research and development actors to the Ministry of Health.

### Future plans

While VICONEL is currently highly representative but slim, there is substantial potential for further integration and thematic expansion. In line with the objective of supporting clinical management, this includes further digitalisation and automation of results sharing with participants, healthcare providers and policymakers, integrating learnings from the VITAL approach. Thematic expansions include (1) optimising integration of point-of-care viral load testing, (2) integration of HIV genotypic resistance test results with the aim of monitoring emerging resistance against newer antiretroviral drugs such as dolutegravir, (3) expansion to diagnostic data on co-morbidities including tuberculosis and (risk factors for) cardiovascular diseases, (4) exploring HIV and ageing and (5) follow-up of mother–child pairs in line with targets for elimination of vertical transmission.^23^ Finally, as the cohort grows in scope, we foresee the potential to embed further randomised trials, using innovative designs such as the trials within cohorts (or cohort multiple randomised controlled trial) approach.^24 25^

## supplementary material

10.1136/bmjopen-2024-085404 online supplemental file 1

## Data Availability

Data are available upon reasonable request.
