# Supplementary material for: Cohort profile: the Viral load Cohort North-East Lesotho (VICONEL) from 2016 to 2023 – cohort description, test volumes, predictors of viraemia and the road ahead
Source: BMJ Open. 2025 Feb 6;15(2):e085404. doi: 10.1136/bmjopen-2024-085404 (PMC11800222; doi:10.1136/bmjopen-2024-085404)
Supplement: online supplemental file 1 [file bmjopen-15-2-s001.docx]

# Supplementary Material

**Table S1: Participant characteristics (2016 – 2023).** For each year, cohort participants with at least one viral load test in the respective year are shown. Data refers to the (time point of the) first viral load test of a given individual in the respective year. In the ‘total’ column, data refers to the (time point of the) last available viral load test for each individual. 3TC: lamivudine; ABC; abacavir; AZT: zidovudine; DTG: dolutegravir; IQR: interquartile range; EFV: efavirenz; LPV/r: ritonavir-boosted lopinavir; NVP: nevirapine; TDF: tenofovir disoproxil fumarate.

|  | **2016** | **2017** | **2018** | **2019** | **2020** | **2021** | **2022** | **2023** | **Total (latest viral load within 2016-2023)** |
| --- | --- | --- | --- | --- | --- | --- | --- | --- | --- |
| **N** | **6,409** | **8,666** | **13,492** | **15,216** | **17,439** | **17,695** | **18,530** | **19,295** | **29,380** |
| Year of blood draw for last viral load, median (IQR) | - | - | - | - | - | - | - | - | 2023 (2022-2023) |
| Years from first to last included viral load, median (IQR) | - | - | - | - | - | - | - | - | 3.5 (1.0-5.5) |
| Number of viral loads per individual, median (IQR) | - | - | - | - | - | - | - | - | 4 (2-7) |
| District, n (%)  Butha-Buthe  Mokhotlong | 6409 (100%)  0 (0%) | 8666 (100%)  0 (0%) | 8840 (66%)  4652 (34%) | 10164 (67%)  5052 (33%) | 10626 (61%)  6813 (39%) | 10985 (62%)  6710 (38%) | 11103 (60%)  7427 (40%) | 11738 (61%)  7557 (39%) | 18165 (62%)  11215 (38%) |
| Health facility type, n (%)  Hospital  Health centre  Other | 4480 (70%)  1902 (30%)  27 (0%) | 5168 (60%)  3218 (37%)  280 (3%) | 7074 (52%)  6100 (45%)  318 (2%) | 7623 (50%)  7211 (47%)  382 (3%) | 8053 (46%)  9018 (52%)  368 (2%) | 8039 (45%)  9216 (52%)  440 (2%) | 8373 (45%)  9791 (53%)  366 (2%) | 8660 (45%)  10308 (53%)  327 (2%) | 13452 (46%)  15269 (52%)  659 (2%) |
| Age, median (IQR)^1^ | 42 (33-52) | 41 (32-52) | 42 (33-52) | 42 (33-52) | 42 (33-53) | 43 (34-53) | 44 (35-54) | 44 (35-55) | 42 (33-53) |
| Age and sex, n (%)^2^  Female adult (≥15 years)  Male adult (≥15 years)  Female child (<15 years)  Male child (<15 years) | 4121 (64%)  1949 (30%)  182 (3%)  156 (2%) | 5590 (65%)  2629 (30%)  239 (3%)  206 (2%) | 8534 (63%)  4237 (31%)  360 (3%)  359 (3%) | 9737 (64%)  4749 (31%)  372 (2%)  356 (2%) | 10717 (61%)  5953 (34%)  383 (2%)  385 (2%) | 10916 (62%)  6087 (34%)  351 (2%)  341 (2%) | 11557 (62%)  6346 (34%)  318 (2%)  309 (2%) | 11975 (62%)  6722 (35%)  310 (2%)  287 (1%) | 18511 (63%)  10029 (34%)  439 (1%)  396 (1%) |
| Year of first recorded ART start, median (IQR) |  |  |  |  |  |  |  |  | 2016 (2012-2018) |
| Years since first recorded ART start, median (IQR) | 3.6 (1.6-6.1) | 3.6 (1.5-6.5) | 4.0 (2.0-7.4) | 4.6 (2.5-8.0) | 5.1 (3.0-8.8) | 6.0 (3.4-9.6) | 6.6 (3.9-10.3) | 7.1 (4.0-11.0) | 6.4 (3.2-9.9) |
| ART regimen, n (%)  TDF+3TC+DTG  ABC+3TC+DTG  AZT+3TC+DTG  TDF+3TC+LPV/r  ABC+3TC+LPV/r  AZT+3TC+LPV/r  TDF+3TC+EFV  ABC+3TC+ EFV  AZT+3TC+ EFV  TDF+3TC+NVP  ABC+3TC+ NVP  AZT+3TC+ NVP  Other/unknown | 0 (0%)  0 (0%)  0 (0%)  43 (1%)  76 (1%)  84 (1%)  3933 (61%)  336 (5%)  932 (15%)  397 (6%)  34 (1%)  549 (9%)  25 (0%) | 0 (0%)  0 (0%)  0 (0%)  74 (1%)  115 (1%)  144 (2%)  5788 (67%)  409 (5%)  1073 (12%)  412 (5%)  30 (0%)  593 (7%)  28 (0%) | 0 (0%)  0 (0%)  0 (0%)  113 (1%)  207 (2%)  260 (2%)  9215 (68%)  562 (4%)  1684 (12%)  477 (4%)  36 (0%)  908 (7%)  30 (0%) | 195 (1%)  10 (0%)  2 (0%)  129 (1%)  248 (2%)  309 (2%)  10689 (70%)  605 (4%)  1594 (10%)  472 (3%)  29 (0%)  901 (6%)  33 (0%) | 7912 (45%)  623 (4%)  141 (1%)  123 (1%)  260 (1%)  255 (1%)  7121 (41%)  233 (1%)  421 (2%)  81 (0%)  7 (0%)  239 (1%)  23 (0%) | 15384 (87%)  954 (5%)  232 (1%)  100 (1%)  252 (1%)  227 (1%)  417 (2%)  39 (0%)  40 (0%)  11 (0%)  4 (0%)  24 (0%)  11 (0%) | 16545 (89%)  966 (5%)  223 (1%)  98 (1%)  231 (1%)  198 (1%)  193 (1%)  18 (0%)  23 (0%)  4 (0%)  1 (0%)  17 (0%)  13 (0%) | 17526 (91%)  1138 (6%)  241 (1%)  62 (0%)  114 (1%)  140 (1%)  45 (0%)  4 (0%)  4 (0%)  1 (0%)  1 (0%)  2 (0%)  17 (0%) | 22167 (75%)  1344 (5%)  309 (1%)  141 (0%)  202 (1%)  253 (1%)  3903 (13%)  215 (1%)  426 (1%)  132 (0%)  19 (0%)  233 (1%)  36 (0%) |

^1^ Missing for one in 2016, one in 2017, one in 2018, two in 2019, one in 2020, and three in the total.

^2^ Missing for one in 2016, two in 2017, two in 2018, two in 2019, one in 2020, one in 2023, and five in the total.


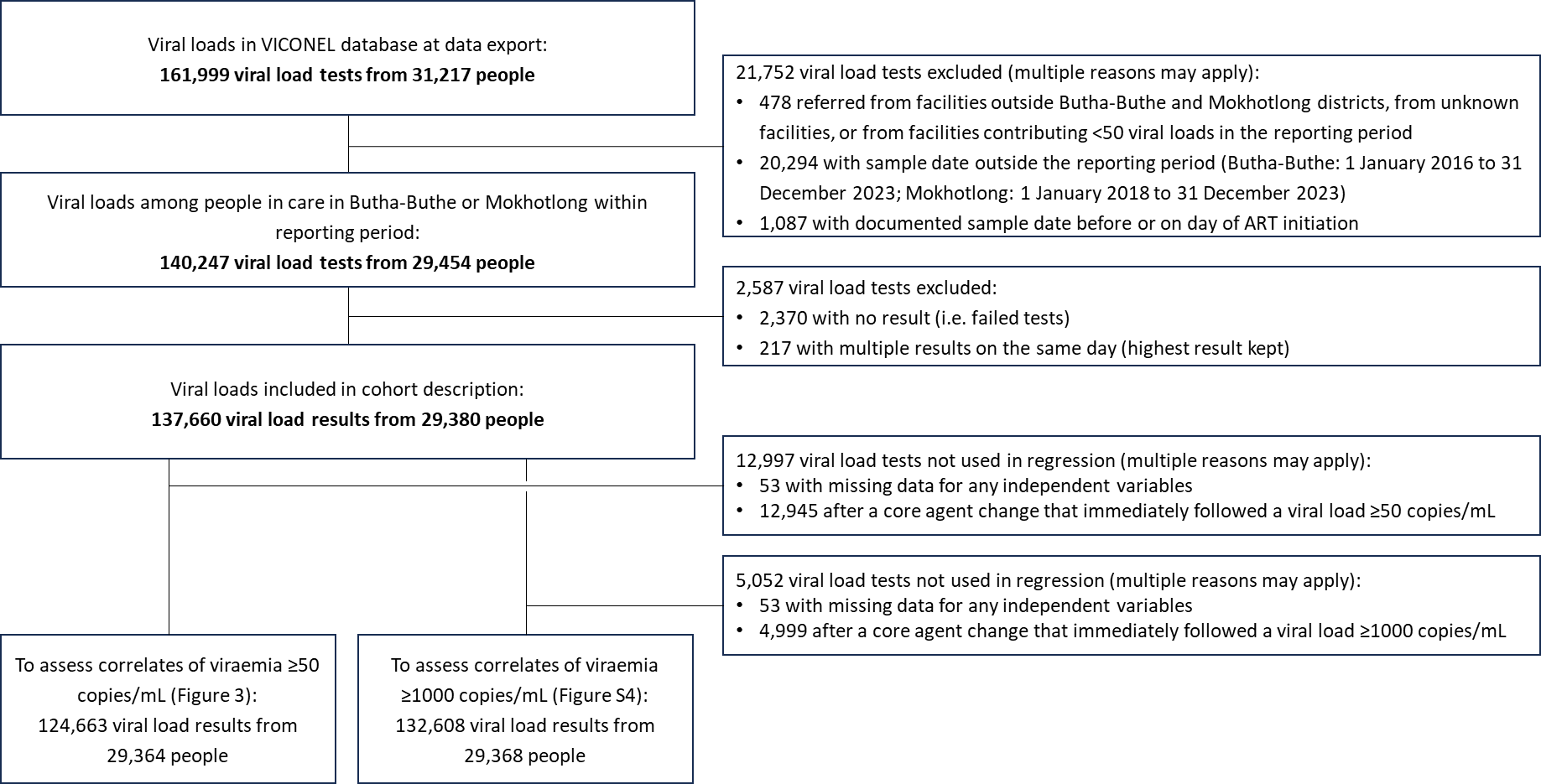


**Figure S1: Flow chart with inclusion criteria.**


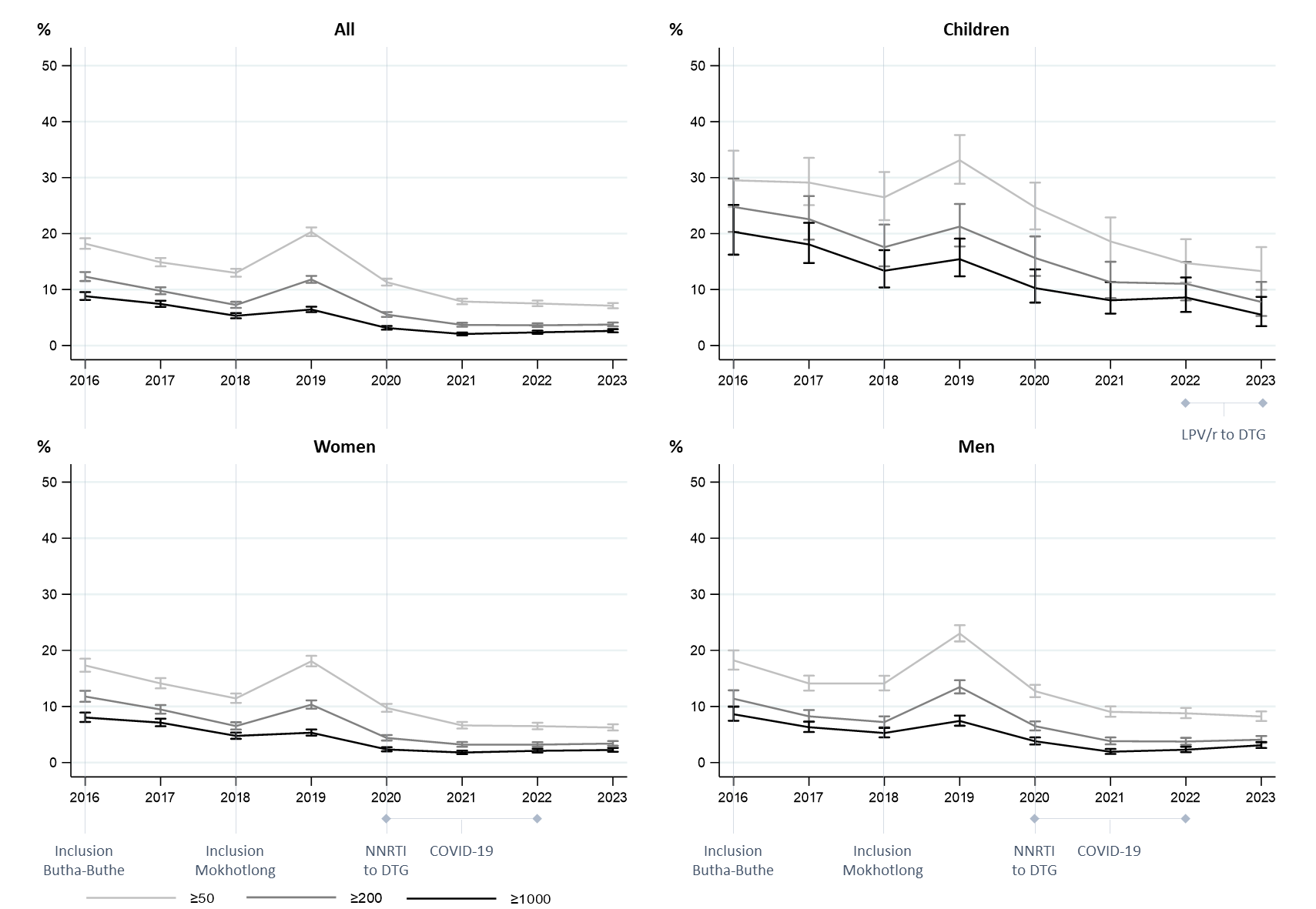


**Figure S2:** **Proportion with viraemia above various thresholds (in copies/mL) over time in Butha-Buthe district.** The first viral load result of any individual in a given year is considered. The denominator corresponds to the number of individuals receiving at least one viral load test in a given year. Bars indicate 95% confidence intervals. DTG: dolutegravir; LPV/r: ritonavir-boosted lopinavir; NNRTI: non-nucleoside reverse transcriptase inhibitor.


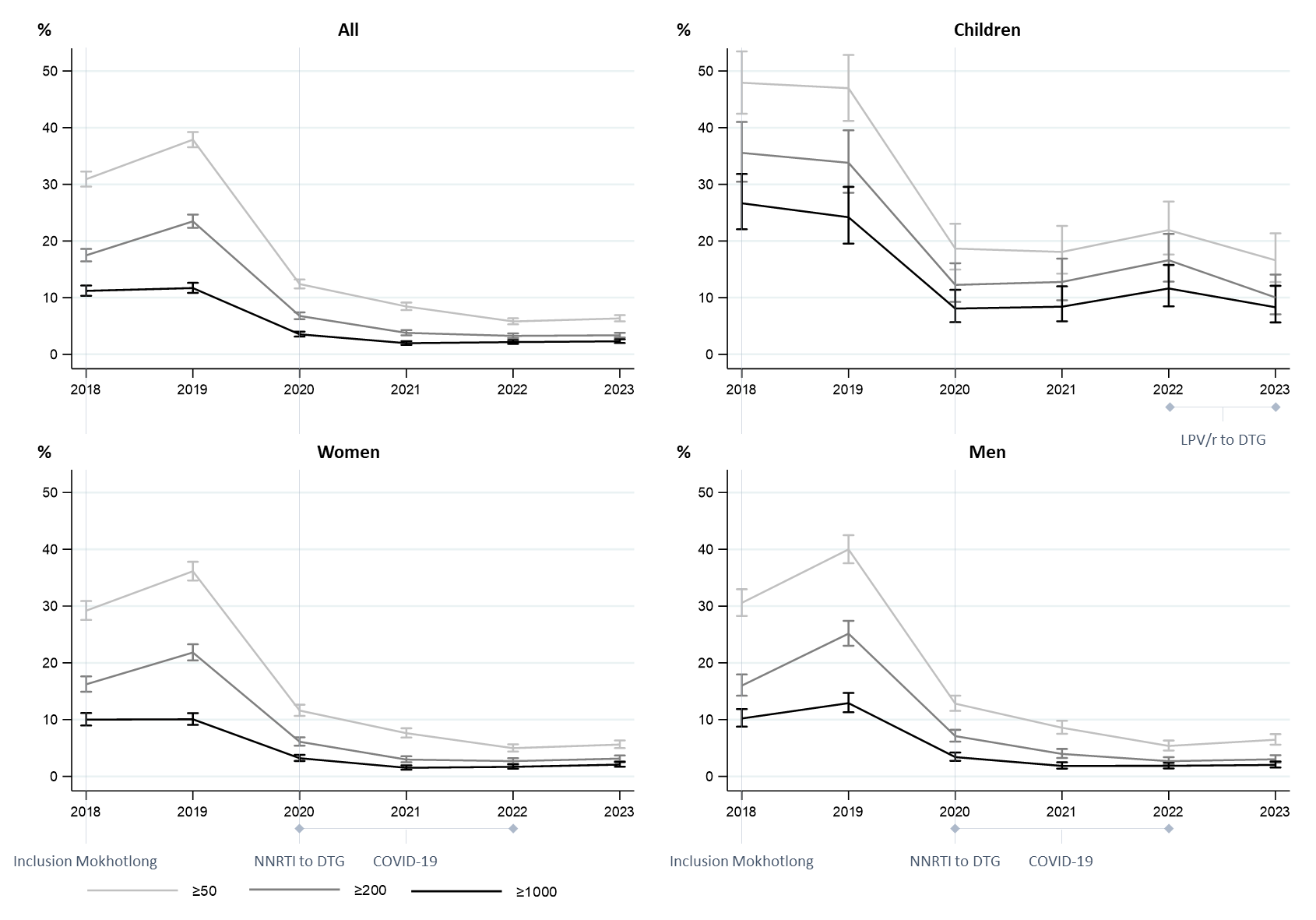


**Figure S3: Proportion with viraemia above various thresholds (in copies/mL) over time in Mokhotlong district.** Mokhotlong district became part of the cohort from 2018. The first viral load result of any individual in a given year is considered. The denominator corresponds to the number of individuals receiving at least one viral load test in a given year. Bars indicate 95% confidence intervals. DTG: dolutegravir; LPV/r: ritonavir-boosted lopinavir; NNRTI: non-nucleoside reverse transcriptase inhibitor.

**Figure S4: Factors associated with a viral load result ≥1,000 copies/mL in a logistic regression with mixed effect on the participant level (N=** **132,608).** Odds ratios and 95% confidence intervals of a given viral load result being ≥1,000 copies/mL are indicated for unadjusted and adjusted analysis. The dotted line at 1 indicates equality of odds. ART: antiretroviral therapy; INSTI: integrase strand transfer inhibitor; PI: protease inhibitor; NNRTI: non-nucleoside reverse transcriptase inhibitor.
