# Supplementary material for: Cohort profile: the Viral load Cohort North-East Lesotho (VICONEL) from 2016 to 2023 – cohort description, test volumes, predictors of viraemia and the road ahead
Source: BMJ Open. 2025 Feb 6;15(2):e085404. doi: 10.1136/bmjopen-2024-085404 (PMC11800222; doi:10.1136/bmjopen-2024-085404)
Supplement: Supplementary file 2 [file bmjopen-2024-085404.reviewer_comments.pdf]

PEER REVIEW HISTORY

BMJ Open publishes all reviews undertaken for accepted manuscripts. Reviewers are asked to complete a checklist review form and are provided with free text boxes to elaborate on their assessment. These free text comments are reproduced below.

ARTICLE DETAILS

Title (Provisional)

Cohort profile: the Viral load Cohort North-East Lesotho (VICONEL) from 2016 to 2023 – cohort description, test volumes, predictors of viraemia, and the road ahead

Authors

Brown, Jennifer Anne; Motaboli, Lipontso; Lerotholi, Malebanye; Kohler, Maurus; Hänggi, Kathrin; Mokete, Moliehi; Chakela, Makobefo Gladys; Kao, Mpho; Kopo, Mathebe; Mokebe, Moleboheng; Urda, Lorena; Nsakala, Bienvenu Lengo; Lukau, Blaise; Ayakaka, Irene; Amstutz, Alain; Ehmer, Jochen; Klicpera, Anna; Klimkait, Thomas; Glass, Tracy; Muhairwe, Josephine; Chammartin, Frédérique; Tschumi, Nadine; Labhardt, Niklaus Daniel D

VERSION 1 - REVIEW

**Reviewer**	**1**
**Name**	**Carlander, Christina**
**Affiliation**	**Karolinska University Hospital**
**Date**	**11-May-2024**
**COI**	**None**

This Cohort profile paper describes the Viral load Cohort North-East Lesotho (VICONEL) from 2016 to 2022. The paper describes the cohort composition, test numbers, treatment outcomes and factors associated with viremia. VICONEL aims to support clinical decisions using a dashboard that flags persons with viremia. The cohort includes 60% women (unlike many European/US cohort where the majority are usually male). Over 27 000 people were included of whom >95% had viral suppression rates <1000 copies, and 7% a viral load>50. Sex, time since ART, district, and type of ART were all associated with viremia. The paper is easy to read and gives a good over-view of the cohort. I have a few comments below.

Abstract: Fine. No comments

Introduction: Fine. No comments

Cohort description:

Q1: I suggest adding a flow chart of the inclusion and exclusion process.

Findings to date:

Q2. Line 262. It is described that self-reported symptoms have been used in reports from the cohort. This is not mentioned among data collected, perhaps this could be added? Or if not seen as part of VICONEL perhaps skip?

Future plans: Fine. No comments

References: Fine.

**Reviewer**	**2**
**Name**	**Kassaye, Seble**
**Affiliation**	**Georgetown University, Georgetown University**
**Date**	**24-Aug-2024**
**COI**	**None**

This is a well written manuscript that describes findings from an prospective open cohort of persons receiving care HIV care and have had HIV VL testing as part of their routine care. Viremia outcomes were assessed based on routinely performed HIV viral load assessments (decentralized laboratory-based (2016 and onwards) and since 2020 the addition of near point of care using the Cepheid GenXpert HIV VL platform at 3 sites), from 2016-2022 in 23 participating clinical settings within two districts in Lesotho. The clinic sites include hospitals (3), clinics (private (1) and health centers (19, one of which is for mine workers). HIV VL testing guidelines call for VL assessments at 6, 12, and then annual testing for adults, and semi-annual assessments for children and adolescents. Relevant to this study is the ART regimen that was in use during the study period that included an NNRTI-based therapy until 2019 when the rollout of dolutegravir-based treatment began.

Overall findings
Of the 27,472 persons with HIV included in the analysis who had VL testing since 2016, only 17K had testing performed in 2022.

The investigators report descriptive statistics for persons with HIV who had VL testing in each year since 2016, providing the reader with some perspective on the service delivery rollout for VL testing. Women remained above 60% of those receiving testing throughout the study period, and DTG based therapy was at 88% in the last year reported in 2022. The median follow up period was ~ 3 years. Viral suppression below 1000 c/mL exceeded 90% since the start of the monitoring period, and remained above 95% from 2020 onward. The investigators also report lower viral suppression rates among children. Mixed effects modeling identified geographic variations that may point to opportunities for improved service delivery and interventions to support treatment adherence in those locations. The finding of improved viral suppression outcomes with INSTI-based therapy supports guideline recommendations for ART use.

Beyond the clinical findings reported, this manuscript provides a summary of laboratory testing capacity and rollout of viral load testing to support clinical service delivery and provides a model that may be replicable in other settings. The manuscript also exemplifies the utility of generating analyzable data in a clinical setting to inform programmatic improvements.

Points for clarification:

1. The statement is made that viral suppression among adults remained below 90% consistently; the investigators should also provide information about what proportion of persons with HIV had HIV VL below 1000 at all viral load assessments? This may differ from an analysis of proportion with viral suppression at each discrete time point.
2. What proportion of persons had VL testing performed as recommended by guidelines – 6, 12 months and annual thereafter. If this information is not available, it would be helpful to include the average number and distribution of VL assessments for individuals.
3. What proportion of children had VL < 1000 c/mL in each time period? The statement provided indicates that the proportion was lower than adults, but a figure should be provided.
4. Are the investigators able to provide any information on how viral load data are used once available – are results provided to patients and at what time period. How soon after viral load indicating viremia are persons contacted and what actions are taken related to the viral load findings.
5. The investigators mention that samples are collected and stored as part of this program. Has viral resistance testing been performed on these samples from persons with demonstrated viremia? This would be helpful to know and may have implications when considering potential use of cabotegravir-based long acting ART.
6. Did the investigators identify differences in viremia outcomes by type of health care setting – hospital vs clinical center – for adults? Among children?
7. The manuscript as currently formatted does not include a discrete discussion section. This would provide an opportunity for the investigators to highlight relevant components from the currently included section under the heading "Review of the cohort's broader findings to date" in a way that is more targeted to the specific questions and findings that are the focus of this manuscript. Other components that are shared in this section may be more appropriate in the background section when describing the cohort.

VERSION 1 - AUTHOR RESPONSE

Reviewer 1

Comments to the author

R1.0: This Cohort profile paper describes the Viral load Cohort North-East Lesotho (VICONEL) from 2016 to 2022. The paper describes the cohort composition, test numbers, treatment outcomes and factors associated with viremia. VICONEL aims to support clinical decisions using a dashboard that flags persons with viremia. The cohort includes 60% women (unlike many

European/US cohort where the majority are usually male). Over 27 000 people were included of whom >95% had viral suppression rates <1000 copies, and 7% a viral load>50. Sex, time since ART, district, and type of ART were all associated with viremia. The paper is easy to read and gives a good over-view of the cohort. I have a few comments below.

Reply: *Thank you for this summary and evaluation of our cohort profile.*

Comments by section

R1.1: Abstract: Fine. No comments.

R1.2: Introduction: Fine. No comments

R1.3 (Q1): Cohort description: I suggest adding a flow chart of the inclusion and exclusion process.

Reply: *We agree and have added a corresponding figure to the supplement (new Figure S1).*

R1.4 (Q2): Findings to date: Line 262. It is described that self-reported symptoms have been used in reports from the cohort. This is not mentioned among data collected, perhaps this could be added? Or if not seen as part of VICONEL perhaps skip?

Reply: *Thank you for this comment. Indeed, these findings stem from a nested study within VICONEL as self-reported symptom data is not routinely available in VICONEL. Approval and consenting procedures for this nested study are described in the cited publication. We now describe this more clearly (line 294):*

> *"In a nested study within VICONEL, we observed no negative changes in self-reported symptoms among ~~For~~ adults changing from efavirenz- to dolutegravir-based ART, ~~we observed no negative impacts of changing to dolutegravir on self-reported symptoms,~~ with potential moderate improvements observed for questions relating to depressed mood, anxiety, and nightmares or strange or vivid dreams[10]."*

R1.6: Future plans: Fine. No comments

R1.7: References: Fine.

Reviewer 2

Comments to the author and overall findings

R2.0:

Comments to the author

This is a well written manuscript that describes findings from an prospective open cohort of persons receiving care HIV care and have had HIV VL testing as part of their routine care.

Viremia outcomes were assessed based on routinely performed HIV viral load assessments (decentralized laboratory-based (2016 and onwards) and since 2020 the addition of near point of care using the Cepheid GenXpert HIV VL platform at 3 sites), from 2016-2022 in 23 participating clinical settings within two districts in Lesotho. The clinic sites include hospitals (3), clinics (private (1) and health centers (19, one of which is for mine workers). HIV VL testing guidelines call for VL assessments at 6, 12, and then annual testing for adults, and semi-annual assessments for children and adolescents. Relevant to this study is the ART regimen that was in use during the study period that included an NNRTI-based therapy until 2019 when the rollout of dolutegravir-based treatment began.

Overall findings

Of the 27,472 persons with HIV included in the analysis who had VL testing since 2016, only 17K had testing performed in 2022.

The investigators report descriptive statistics for persons with HIV who had VL testing in each year since 2016, providing the reader with some perspective on the service delivery rollout for VL testing. Women remained above 60% of those receiving testing throughout the study period, and DTG based therapy was at 88% in the last year reported in 2022. The median follow up period was ~ 3 years. Viral suppression below 1000 c/mL exceeded 90% since the start of the monitoring period, and remained above 95% from 2020 onward. The investigators also report lower viral suppression rates among children. Mixed effects modeling identified geographic variations that may point to opportunities for improved service delivery and interventions to support treatment adherence in those locations. The finding of improved viral suppression outcomes with INSTI-based therapy supports guideline recommendations for ART use.

Beyond the clinical findings reported, this manuscript provides a summary of laboratory testing capacity and rollout of viral load testing to support clinical service delivery and provides a model that may be replicable in other settings. The manuscript also exemplifies the utility of generating analyzable data in a clinical setting to inform programmatic improvements.

Reply: *Thank you for this evaluation of the cohort profile and the clear summary of our key findings. As data through 2023 is now available, we have updated our report with 2023 data.*

Points for clarification

R2.1: The statement is made that viral suppression among adults remained below 90% consistently; the investigators should also provide information about what proportion of persons with HIV had HIV VL below 1000 at all viral load assessments? This may differ from an analysis of proportion with viral suppression at each discrete time point.

Reply: *We have added the following sentence (line 254):*

> *"Among all participants, regardless of the number of recorded viral load results, all viral load results were <50 copies/mL for 18,325/29,380 (62 %), at least one was ≥50 copies/mL and all were <1000 copies/mL for 7,024/29,380 (24%), and at least one result was ≥1000 copies/mL for 4,031/29,380 (14%)."*

R2.2: What proportion of persons had VL testing performed as recommended by guidelines – 6, 12 months and annual thereafter. If this information is not available, it would be helpful to include the average number and distribution of VL assessments for individuals.

Reply: *We agree that guideline-adherent VL testing is an important concern. We have slightly extended the results as follows (line 215):*

> *"Median follow-up time from the first to the last ~~available~~ included viral load result was ~~3.0~~3.5 years (IQR 1.0-~~4.6~~5.5); during this time, a median of 4 (IQR 2-7) viral load results were recorded (**Table 1**)."*

We have also added the time from the first to the last viral load result to Table 1, and now show this and the number of viral loads at the top of Table 1.

While VICONEL covers a substantial geographic region and we are able to link people between included facilities, the population in this region is mobile within Lesotho and neighbouring South Africa, complicating longitudinal assessments of guideline-adherent viral load testing. As such, we feel that such analyses are beyond the scope of the present cohort profile. However, we have previously conducted more in-depth analyses of clinical action following detected viraemia among children/adolescents and adults. These findings are referenced in line 285:

> *"First, management of viraemia – i.e., timely follow-up viral load testing with switching to second-line ART if indicated – was assessed among children[5] and adults[4], demonstrating major gaps in the viral load care cascade. Among children and adults with a viral load ≥1,000 copies/mL, only 28% and 25%, respectively, were considered to be managed in a timely manner according to guidelines."*

Similarly, we have recently assessed guideline adherence of viral load testing during pregnancy and postpartum, as well as infant HIV testing. These findings have been submitted and were presented at HIV Glasgow 2024.

R2.3: What proportion of children had VL < 1000 c/mL in each time period? The statement provided indicates that the proportion was lower than adults, but a figure should be provided.

Reply: *We have extended the text slightly as follows (line 244):*

> *"Throughout the observation period, viral suppression ~~rates~~ to <1,000 copies/mL among children ~~have~~ has been substantially lower than among adults at around 80-82% from 2016-2019 and around 90-93% since 2020~~;~~. ~~this~~ This is consistent with reported challenges in achieving viral suppression among children and global reports[14,20]."*

Please see Figure 2, Figure S2, and Figure S3 (upper right panel for all).

R2.4: Are the investigators able to provide any information on how viral load data are used once available – are results provided to patients and at what time period. How soon after viral load indicating viremia are persons contacted and what actions are taken related to the viral load findings.

Reply: *Unfortunately, we do not have routine data on the time needed for results to reach treating clinicians or patients. However, we have investigated time to clinical action in previous publications, identifying substantial gaps (line 285):*

> *"First, management of viraemia – i.e., timely follow-up viral load testing with switching to second-line ART if indicated – was assessed among children[5] and adults[4], demonstrating major gaps in the viral load care cascade. Among children and adults with a viral load ≥1,000 copies/mL, only 28% and 25%, respectively, were considered to be managed in a timely manner according to guidelines."*

The VITAL cohort nested within VICONEL, which we describe in line 317-323, will also shed some light on these questions. We expect first trial findings to become available in 2025.

R2.5: The investigators mention that samples are collected and stored as part of this program. Has viral resistance testing been performed on these samples from persons with demonstrated viremia? This would be helpful to know and may have implications when considering potential use of cabotegravir-based long acting ART.

Reply: *Genotypic resistance testing is not commonly implemented in routine care. We have added the following information to the "Procedures" section under "Methods" (line 140):*

> *"Specifically, viraemia ≥1000 copies/mL or, since 2022, ≥50 copies/mL should trigger several sessions of enhanced adherence counselling followed by repeat viral load testing. For NNRTI-based first-line ART, a switch to second-line ART was indicated in case of sustained viraemia. For sustained viraemia with PI- or INSTI-based ART, national guidelines recommend consulting the ART advisory committee and considering genotypic resistance testing[17,18]. However, genotypic resistance testing and subsequent treatment switches require centralised approval, are relatively uncommon, and resistance results are currently not routinely transferred to the VICONEL database."*

Nevertheless, we have conducted resistance testing in a nested study using stored samples among consenting individuals, feeding treatment-relevant findings back to clinicians. We have slightly edited the description of this study for clarity (line 305):

> *"among ~~the few~~ 85 participants ~~with~~ who had persistent and/or recurring viraemia including at least one viral load ≥500 copies/mL at least 18 months after starting dolutegravir, consented to further use of their stored samples, and had available samples in the biobank that were successfully sequenced, dolutegravir resistance was observed for 8 ~~approx. 10~~ (9%)[23]. All eight participants with dolutegravir resistance were in care at health centres (as opposed to hospitals)."*

R2.6: Did the investigators identify differences in viremia outcomes by type of health care setting – hospital vs clinical center – for adults? Among children?

Reply: *We did not observe an association of receiving care in a hospital (note that this would typically be outpatient care). We acknowledge that this is the only variable that had not been explicitly mentioned in text (as no association was observed) and that this was difficult to see in Figure 3. We now write (line 267):*

> *"No association with viraemia was observed for receiving care in a hospital (vs health centre or other facility type; aOR 0.95, 95% CI 0.89-1.00)."*

The upper bound of the 95% confidence interval is rounded down (1.003). Similarly, we did not observe an association among children (aOR 0.88, 95% CI 0.69-1.13) or adults (aOR 0.95, 95% CI 0.90-1.01).

However, we have seen differences in care and/or treatment outcomes in previous studies within VICONEL that we reference here. We have now added the following information.

Line 285:

> *"First, management of viraemia – i.e., timely follow-up viral load testing with switching to second-line ART if indicated – was assessed among children[5] and adults[4], demonstrating major gaps in the viral load care cascade. Among children and adults with a viral load ≥1,000 copies/mL, only 28% and 25%, respectively, were considered to be managed in a timely manner according to guidelines. Compared with adult participants in care in hospitals, adults in health centres were more likely to have a viral load ≥1,000 copies/mL at the follow-up viral load test (64% vs 44%) and less likely to be switched to second-line therapy in case of sustained viraemia[4]. In children, delays between a first*

elevated viral load and confirmatory viral load testing were more pronounced in health centres than in hospitals[5].”

Line 305:

“among ~~the few~~ 85 participants ~~with~~ who had persistent and/or recurring viraemia including at least one viral load ≥500 copies/mL at least 18 months after starting dolutegravir, consented to further use of their stored samples, and had available samples in the biobank that were successfully sequenced, dolutegravir resistance was observed for 8 ~~approx. 10~~ (9%)[23]. All eight participants with dolutegravir resistance were in care at health centres (as opposed to hospitals).”

R2.7: The manuscript as currently formatted does not include a discrete discussion section. This would provide an opportunity for the investigators to highlight relevant components from the currently included section under the heading “Review of the cohort’s broader findings to date” in a way that is more targeted to the specific questions and findings that are the focus of this manuscript. Other components that are shared in this section may be more appropriate in the background section when describing the cohort.

Reply: *We acknowledge that the cohort profile layout differs from the classical format for manuscripts reporting original research. We have formatted the manuscript to align with the journal’s specific guidance for cohort profiles as a separate article type.*

VERSION 2 - REVIEW

**Reviewer**	**2**
**Name**	**Kassaye, Seble**
**Affiliation**	**Georgetown University, Georgetown University**
**Date**	**11-Jan-2025**
**COI**	

All prior review recommendations have been addressed. The manuscript is well written and findings are clearly reported. No additional recommended revisions.
